# Status of nutrition labeling knowledge, attitude, and practice (KAP) of residents in the community and structural equation modeling analysis

**DOI:** 10.3389/fnut.2023.1097562

**Published:** 2023-04-17

**Authors:** Yinxia Liao, Jianjun Yang

**Affiliations:** ^1^School of Public Health and Management, Ningxia Medical University, Yinchuan, Ningxia, China; ^2^Ningxia Key Laboratory of Environmental Factors and Chronic Disease Control, Yinchuan, Ningxia, China

**Keywords:** prepackaged food, nutrition labeling, knowledge-attitude-practice model, residents, structural equation model

## Abstract

**Objective:**

Unhealthy foods were a major contributor to the occurrence of chronic non-communicable diseases. The promotion of nutrition labeling in the community can effectively help residents to choose healthy foods, which plays an important role in the prevention of chronic diseases. However, the public awareness of this measure is not clear. Our study used a structural equation model based on the KAP theory to analyze the interaction mechanisms among knowledge, attitude, and practice and aimed to evaluate the relationships among nutrition knowledge, attitude, and practice of residents, which can provide the basis of policy formulation for nutrition education and behavior intervention.

**Methods:**

We carried out a cross-sectional study from May 2022 to July 2022 in the “Community Health Service Center”, and each “Community Service Station” in Yinchuan use a self-designed questionnaire and convenience sampling to evaluate resident nutrition labeling KAP status. This study adopted the structural equation modeling approach to analyze a survey of Chinese individuals through the cognitive processing model, interrelated nutrition knowledge, nutrition label knowledge, attitude, and practice.

**Results:**

According to the principle of sample size estimation, a total of 636 individuals were investigated, with the ratio of male to female being 1:1.2. The average score of community residents' nutrition knowledge was 7.48 ± 3.24, and the passing rate was 19.4%. Most residents had a positive attitude toward nutrition labeling, but the awareness rate was only 32.7% and the utilization rate was 38.5%. Univariate analysis showed that women had higher knowledge scores than men (*p* < 0.05), and young people had higher scores than older adults (*p* < 0.05), and the difference was significant. Based on the KAP structural equation model (SEM), residents' nutrition knowledge will directly affect their attitude toward nutrition labeling. Attitude played a greater role as an indirect effect between knowledge and behavior, while trust limits residents' practice of nutrition labeling and then affects their practice. It could be explained that nutrition knowledge was the prerequisite for label reading behavior, and attitude was the intermediary effect.

**Conclusion:**

The nutrition knowledge and nutrition labeling knowledge of respondents hardly directly support the practice of nutrition labeling, but it can influence the use behavior by forming a positive attitude. The KAP model is suitable for explaining residents' use of nutrition labeling in the region. Future research should focus on better understanding the motivations of residents to use nutrition labeling and the opportunity to use nutrition labeling in real-life shopping settings.

## 1. Introduction

In recent years, fast foods, take-out foods, and prepackaged foods have become increasingly popular, with a rapid increase in the consumption rate of them (1–3), among which, the consumption rate of prepackaged foods in China has reached 59.8% (4). The poor cooking methods of take-out foods and fast foods caused a large accumulation of unhealthy ingredients in the body, including fat, salt, and sugar (1, 5). Prepackaged foods (including puffed food, beverage, pickled canned food, and leisure food) are generally high in energy, fat, and sodium and low in protein and dietary fiber (6, 7). Whether it is fast foods, take-out foods, or prepackaged foods, its rising consumption rate and accumulation of unhealthy nutrients are the key factors in causing the high incidence of chronic diseases such as obesity and diabetes (8, 9). Diet-related diseases have become more common because of changes in lifestyle and food habits, but researchers have also established that dietary modifications significantly reduce the risk of diseases (10). Individual food choices and eating behaviors are influenced by many interrelated factors which affect the results of nutrition-related public health interventions. To improve the adverse health effects of this situation, recommendations and interventions have been implemented across the globe. Nutrition labeling plays an active role as a dietary strategy as recommended by the WHO (11). In the face of increasing diet-related chronic diseases, many countries have initiated steps to include nutrition labeling on prepackaged food packets and in restaurant menus to standardize the management of nutrition labeling (12).

Nutrition labeling is not only an information tool to interpret the nutrient content and function of food but also a strategy against overweight and obesity (13), which plays a critical role in promoting healthy eating habits. Petimar et al. found that the calorie menu labeling was associated with an immediate decrease of 60 calories per transaction or 4% of total calories purchased (14). A meta-analysis expressed that food labeling decreased consumer intakes of energy by 6.6%, total fat by 10.6%, artificial trans fat by 64.3%, sodium by 8.9%, and other unhealthy dietary options by 13.0% while increasing vegetable consumption by 13.5% (15). The implementation of nutrition labeling and sugar labeling can contribute to the lower risk of cardiovascular diseases and cancer and kidney diseases, thereby reducing the prevalence of chronic diseases and increasing life expectancy (16, 17). In addition, under the Nutrition Labeling and Education Act (NLEA) promulgated by the United States in 1990, nutrition labeling will be required for all retail food products to facilitate consumers to obtain more nutrition information and maintain healthy dietary practices (18). Although the nutrition labeling system has been introduced in China as early as 1994 (19) and has been revised several times, the practice of nutrition labeling has not been actually promoted until the General Rules of National Prepackaged Food Nutrition Labels (GB 28050-2013) were enacted in 2013 (20, 21). Since then, the labeling rate of nutrition labeling in prepackaged foods has been significantly improved (22). Since then, the labeling rate of nutrition labeling in prepackaged foods has been significantly improved. However, the actual utilization of nutrition labeling was in fact lower than that reported (23), possibly because the complex design of nutrition labeling is puzzling, including energy conversion and professional terms description (24, 25). Previous studies have found that the longer a consumer gazed at the nutrition claim, the more likely the product with a nutrition claim was bought (26). It is also reported that consumers who regularly use the nutrition labeling seem to have a higher diet quality (27).

The knowledge, attitude, and practice (KAP) model is a theory to explain an individual healthy behavior (28) and the model figures that there are two key steps to changing behavior: establishing beliefs and changing attitudes. Up to now, the KAP model has been widely applied to health education in the fields of prevention of primary infectious diseases (e.g., schistosomiasis, tuberculosis, malaria, and AIDS) (29–31) and control of chronic diseases (e.g., diabetes and hypertension) (32, 33). However, few studies have explored the relationships among knowledge, attitude, and practice behavior of nutrition labels based on the KAP theory (34–36). The use of nutrition labeling is a dietary self-management behavior and is closely related to their own nutrition knowledge and health beliefs. Therefore, we adopted the KAP model as a framework to explore the relationships between them, and the new findings may contribute to future nutrition education to promote nutrition label use in China.

## 2. Hypotheses of the KAP model

**Figure 1:**
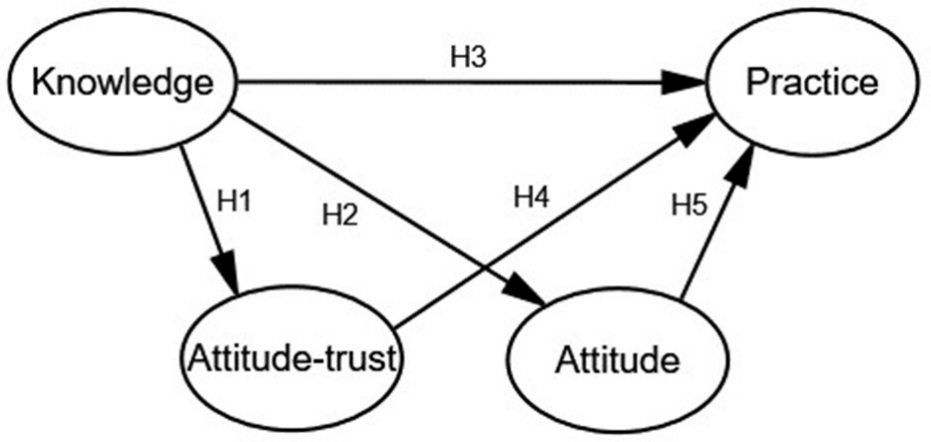
The KAP constructed equation model.

According to the KAP theory, there is a causal relationship among knowledge, attitude, and practice (37). However, KAP are potential variables that are difficult to measure directly. The traditional statistical methods cannot deal with these potential variables effectively, while the structural equation model (SEM) integrates the traditional statistical analysis methods, such as confirmatory factor analysis, path analysis, and multiple regression analysis, leading to a new multivariate statistical technology. It can not only analyze and deal with measurement errors but also analyze the structural relationship between potential variables (38, 39) and directly display the correlation between the variables through the path diagram. In addition, it can also explore the causal relationship between potential variables and quantitatively evaluate the direct and indirect effects of variables (40), as shown in Figure 1.

Knowledge means the ability of understanding and using nutrition information, through education, learning experience, and identifying the nutrition label. Attitude refers to the feeling or opinions of community residents on nutrition labeling in some situations, including credibility, helpfulness, and necessity. Practice refers to the use or application of nutrition labeling by community residents. Based on the KAP model, it is predicted that nutrition knowledge will positively and indirectly affect practice through attitude change, and nutrition knowledge may also directly affect nutrition labeling practice. We put forward the following five assumptions based on the relevant literature on the knowledge, attitude, and practice structure model published by Zeng Y, Kwak C, Zeying H, and Misra R.

Hypothesis 1(H1): Community residents who have higher nutrition knowledge scores are more likely to trust nutrition labeling.
Hypothesis 2(H2): Community residents who have higher nutrition knowledge scores are more likely to have a positive attitude toward labeling.
Hypothesis 3(H3): Community residents who have higher nutrition knowledge scores are more likely to use nutrition labels.
Hypothesis 4(H4): Community residents who have more trust in nutrition labeling are more likely to use it.
Hypothesis 5(H5): Community residents who have a more positive attitude toward nutrition labeling are more likely to use it.

Thus, we attempted to analyze the interactions among community residents' nutrition labeling knowledge, attitude, and practice by using the KAP model to construct a structural equation. Meanwhile, we should also explore residents' cognition and use behaviors of nutrition labeling, as well as the influencing factors so that the resident can have a better understanding of nutrition labels and habits of food choice.

## 3. Materials and methods

### 3.1. Materials

A cross-sectional questionnaire survey was conducted using convenience sampling and anonymously in a community health service center in Yinchuan, Ningxia, from 1 May 2022 to 16 July 2022. Investigators will be rigorously trained before the investigation, and the data collected will be kept strictly confidential by the research team. The data were collected by a combination of online and onsite. The respondents include adults over 18 years old who have lived in the community for a long time (more than one year), excluding residents with serious diseases and unable to communicate. After informed consent was obtained from each participant, questionnaires are distributed and filled out. The sample size calculation is as follows: $n=Z_{1-\text{α}/2}^{2}\times P\times \frac{\left(1-P\right)}{\delta^{2}}$, (where α: significance level, when α= 0.05, Z_1−α/2_ = 1.96, *n*:sample content,δ: allowable error, and *P*:estimation value of population rate π). The average awareness rate of the nutrition label is approximately 40%, that is, *P* = 0.4, α = 0.05, and δ = 0.04, the sample size was expanded by 10% considering non-response, and 636 residents were eventually included.

### 3.2. Methods and collection data

This study is based on KAP model (41). The questionnaire is based on the questionnaire designed by the Center for Disease Control and Prevention (CDC) of China, then revised according to Cui (42) (Cronbach's alpha = 0.967, Kaiser–Meyer–Olkin = 0.960, *p* < 0.005) and Liu (43), and finally verified by expert review. Two pre-surveys were conducted in a small sample of 62 adults, which were revised according to the feedback. We should ensure that the reliability and validity of the final questionnaire were qualified (Cronbach's alpha = 0.922, Kaiser–Meyer–Olkin = 0.887, *p* < 0.001). The questionnaire includes 50 questions in three parts as follows: basic demographic information, nutrition knowledge, and nutrition labeling KAP; each part is relatively independent. The first part includes answering questions such as age, gender, educational level, marital status, occupation, monthly income, self-reported illness or physical condition, and medical and nutrition education, and this part is not scored. The second part includes answering questions about the main effects of core nutrients (such as protein, fat, carbohydrates, and sodium) and the recommended intake of sodium in *Dietary Guidelines for Chinese Residents*. In this part, multiple choice questions (single-choice question), 1 point for the right choice. Multiple choice questions (select one or more answer choices), with 0.5 points for each correct item. The third part includes answering the contents of nutrition labeling, the meaning of NRV, and the types of nutrients that are mandatory to be labeled, with 1 point for the right choice. In this part, the questions about residents' understanding, attitude, trust, and helpfulness of nutrition labeling are evaluated by a five-point Likert scale, ranging from 1 “strongly disagree” to 5 “strongly agree”. The scores are 1, 2, 3, 4, and 5, respectively, which increase in turn.

The number of correct answers to “What are the parts of food nutrition labels” divided by the total number of samples, which is the awareness rate of nutrition labels, expressed as a percentage. Regarding the numerical expressions of credibility, helpfulness, and necessity, we combine “strongly agree” and “agree” as positive, “neither agree nor disagree” as modest, and “strongly disagree” and “disagree” as negative. The higher the score, the higher the residents' understanding of food nutrition labels, the more positive their attitude, and the more willing they are to use nutrition labels when shopping. Data were collected in “A Community Health Service Center” and an online questionnaire platform “Wenjuanwang” (https://www.wenjuan.com/).

## 4. Data analysis methods

Data analysis was performed in three stages. First, the data were analyzed using EpiData3.1 data entry. Second, SPSS 24.0 (IBM, NY, United States) was used for statistical analysis and reliability and validity tests. If the quantitative data were subjected to the normal distribution, it is described by mean standard deviation (mean ± *SD*); On the contrary, it is described by median or interquartile value. If the data submitted to normal distribution and homogeneity of variance, a one-way analysis of variance or chi-square test was used. Otherwise, the Wilcoxon rank-sum test is adopted for comparison. Finally, the KAP structural equation model (SEM) of nutrition labeling for community residents was constructed by using AMOS 24.0 (IBM, NY, United States) software, and the model was revised by Modification Indices. The model fitting was evaluated with χ^2^ -value, GFI (goodness-of-fit index), AGFI (adjusted goodness-of-fit index), TLI (Tucker–Lewis index), CFI (comparative fit index), NFI (normed fit index), IFI (incremental fit index), and RMSEA (root mean square error of approximation). The test level was 0.05, and *p* < 0.05 indicated that the difference was statistically significant.

## 5. Results

### 5.1. Demographic data analysis

The sociodemographic characteristics of the participants are presented in Table 1. A total of 636 people were investigated, including 285 men and 351 women, mean age was approximately 46.8 ± 17.0 years with a minimum age of 18 years and a maximum age of 75 years. The most frequent age group was 35–44 years (21.9%). More than half of the residents have received a high school education or above (67.7%). Residents with a monthly income between 5,000 and 10,000 CNY are the most, account for 34.6%, and with monthly income above 20,000 CNY being the least, accounting for only 1.9%; and 74.7% of the residents are married.

**Table 1:** Sociodemographic characteristics of the whole sample.

**Variables**	**Profile**	***N***	**Percentage%**
Gender	Male	285	44.8
	Female	351	55.2
Age	18–29	139	21.9
	30–39	98	15.4
	40–49	117	18.4
	50–59	103	16.2
	60–69	95	14.9
	70–	84	13.2
Education level	Primary school and below	86	13.5
	Junior school	120	18.9
	High school or technical secondary school	145	22.8
	Junior college or undergraduate	263	41.4
	Postgraduate level and above	22	3.5
Monthly Earning (*yen*)	< 1,500	39	6.1
	1,501–3,000	112	17.6
	3,001–5,000	207	32.5
	5,001–10,000	220	34.6
	10,001–20,000	46	7.2
	≥20,000	12	1.9
marital status	Unmarried	109	17.1
	Married	475	74.7
	Divorced	23	3.6
	Widowed	29	4.6

### 5.2. Knowledge, attitude, and practice of nutrition labeling among residents by different gender

The scores of nutrition labeling knowledge, attitude, and practice of women were higher than those of men. There were no significant differences in genders in understanding nutrition labeling information and technical term descriptions (*p* > 0.05), but there were significant differences in understanding nutritional content, numerical information, and the function of nutritional content (*p* < 0.05). In terms of nutrition labeling attitude, women showed significantly higher scores (*p* < 0.05) in necessity and helpfulness except credibility, compared to men. It indicated that women had richer nutrition labeling knowledge, more positive attitudes toward nutrition labeling, and used nutrition labeling more frequently, which was related to the fact that the frequency of undertaking food purchasing and cooking was higher in women than in men. It might be because women received medical or nutrition-related training more frequently than men, as shown in Table 2.

**Table 2:** Mean scores on knowledge, attitude, and practice scales completed by 636 residents, by gender.

**Variables (Mean ±SD)**	**Total samples**	**Gender**		**t/h**	P^*^
		**Male**	**Female**		
Nutrition knowledge	7.5 ± 3.2	7.1 ± 3.4	7.7 ± 3.1	−2.36	0.019
Understand the information on nutrition labeling	2.9 ± 1.0	2.8 ± 1.0	2.9 ± 1.0	−1.48	0.141
Understand technical term description	2.3 ± 1.1	2.2 ± 1.1	2.4 ± 1.2	−1.91	0.056
Understand the nutrients.	3.1 ± 1.2	2.9 ± 1.1	3.3 ± 1.2	−4.19	< 0.001
Understand the numerical information and units	2.8 ± 1.3	2.5 ± 1.3	3.0 ± 1.3	−4.75	< 0.001
Understand the function of nutrients	3.2 ± 1.2	3.0 ± 1.2	3.4 ± 1.2	−3.69	< 0.001
Nutrition labels are credible	3.2 ± 0.9	3.1 ± 0.9	3.2 ± 0.9	−0.88	0.379
Nutrition Facts Table are credible	3.1 ± 0.9	3.0 ± 0.9	3.1 ± 0.9	−1.33	0.184
Nutrition claims are credible	3.1 ± 0.9	3.0 ± 0.9	3.1 ± 0.9	−1.13	0.258
Nutrition function claims are credible	3.2 ± 0.9	3.2 ± 0.9	3.1 ± 0.9	0.26	0.798
Nutrition labels are helpful	3.5 ± 1.0	3.3 ± 1.0	3.6 ± 1.0	−3.00	0.003
Nutrition labels are necessary (Media)	4.0(4.0~5.0)	3.0(3.0~5.0)	4.0(4.0~5.0)	13.24	< 0.001
Use nutrition labels when shopping	3.2 ± 1.1	2.9 ± 1.1	3.3 ± 1.0	−4.36	< 0.001
Check types or contents of nutrients	3.3 ± 1.2	2.9 ± 1.2	3.4 ± 1.1	−4.58	< 0.001
Check Energy	3.1 ± 1.2	2.8 ± 1.2	3.3 ± 1.2	−5.02	< 0.001
Check NRV%	2.8 ± 1.2	2.6 ± 1.2	3.0 ± 1.2	−4.36	< 0.001
Check nutrition claims	3.2 ± 1.2	3.0 ± 1.2	3.4 ± 1.1	−4.04	< 0.001
Check Nutrient function claims	3.3 ± 1.2	3.0 ± 1.2	3.3 ± 1.1	−3.15	0.002

^*^student's *t*-test (T) or Kruskal–Wallis test (H), *p* < 0.05 is considered statistically significant.

### 5.3. Knowledge, attitude, and practice of nutrition labeling among residents by different economic conditions

The residents with a higher monthly household income had higher nutrition knowledge scores, and the difference was significant (*p* < 0.05). The residents with better economic conditions scored higher than those with poorer economic conditions in nutrition labeling knowledge, attitude, and practice, with a significant difference (*p* < 0.05). However, whether residents checked nutrition claims and nutrient function claims during shopping was not significantly different with respect to their socioeconomic status (*p* > 0.05), as shown in Table 3.

**Table 3:** Mean scores on knowledge, attitude, and practice scales completed by 636 residents, by income.

**Variables (mean ±SD)**	**Total samples**	**Income**			***h***	P^*^
		**Low**	**Medium**	**High**		
Nutrition knowledge	7.5 ± 3.2	6.0 ± 2.9	7.8 ± 3.2	9.2 ± 3.2	47.32	< 0.001
Understand the information on nutrition labeling	2.9 ± 1.0	2.6 ± 1.0	3.0 ± 1.0	3.1 ± 0.9	21.79	< 0.001
Understand technical term description	2.3 ± 1.1	2.1 ± 1.1	2.3 ± 1.1	2.4 ± 1.3	6.61	0.037
Understand the nutrients.	3.1 ± 1.2	2.7 ± 1.2	3.2 ± 1.1	3.6 ± 1.0	35.18	< 0.001
Understand the numerical information and units	2.8 ± 1.3	2.4 ± 1.3	2.9 ± 1.3	3.2 ± 1.3	18.81	< 0.001
Understand the function of nutrients	3.2 ± 1.2	2.9 ± 1.3	3.3 ± 1.2	3.7 ± 1.1	19.63	< 0.001
Nutrition labels are credible	3.2 ± 0.9	3.0 ± 0.9	3.2 ± 0.8	3.3 ± 0.8	13.35	0.001
Nutrition Facts Table are credible	3.1 ± 0.9	2.9 ± 0.9	3.1 ± 0.9	3.3 ± 1.1	11.05	0.004
Nutrition claims are credible	3.1 ± 0.9	2.9 ± 0.9	3.1 ± 0.9	3.2 ± 1.0	6.89	0.032
Nutrition function claims are credible	3.2 ± 0.9	3.0 ± 1.0	3.2 ± 0.9	3.4 ± 1.0	11.08	0.004
Nutrition labels are helpful	3.5 ± 1.0	3.2 ± 1.2	3.5 ± 1.0	3.8 ± 1.0	15.17	0.001
Nutrition labels are necessary (Media)	4.0(4.0~5.0)	4.0(3.0~5.0)	4.0(4.0~5.0)	4.0(4.0~5.0)	8.31	0.016
Use nutrition labels when shopping	3.2 ± 1.1	2.9 ± 1.2	3.2 ± 1.1	3.5 ± 1.0	16.27	< 0.001
Check types or contents of nutrients	3.2 ± 1.2	2.9 ± 1.3	3.2 ± 1.1	3.6 ± 1.1	12.42	0.002
Check Energy	3.1 ± 1.2	2.8 ± 1.3	3.1 ± 1.2	3.5 ± 1.2	14.54	0.001
Check NRV%	2.8 ± 1.2	2.6 ± 1.2	2.9 ± 1.2	3.0 ± 1.2	6.25	0.044
Check nutrition claims	3.2 ± 1.2	3.1 ± 1.3	3.2 ± 1.1	3.4 ± 1.1	4.43	0.109
Check Nutrient function claims	3.2 ± 1.2	3.0 ± 1.3	3.3 ± 1.1	3.2 ± 1.2	5.25	0.073

Low: Monthly income < 3,000 yuan, Medium: Monthly income is between 3,000 and 10,000 yuan, High: The monthly income is more than 10,000 yuan. Criteria for division: According to the “People's Republic of China (PRC) 2022 National Economic and Social Development Statistics Bulletin” published by the National Bureau of Statistics of China. The national per capita disposable income is 36,883 yuan, and the average monthly income is approximately 3,000 yuan. ^*^Student's t-test (T) or Kruskal–Wallis test (H), p < 0.05 is considered statistically significant.

Only 25.5% of the residents could understand the information on the nutrition labeling, with the worst understanding of the description of professional terms and the better understanding of the role of nutrients. In total, 76.1% considered it necessary to implement nutrition labeling, and 45.8% of the residents would check the nutrition labeling, but only 38.5% of them said that the nutrition labeling could affect their shopping behavior. Residents still had an inherent distrust on the authenticity of nutrition labeling, with 20.5% of the residents considered that the nutrition labeling was generally untrustworthy, 23.6% considered that the nutrition table was inaccurate, 23.0% considered that the nutrition claims were untrue, and 21% considered that the nutrition function claims were untrustworthy. It can be seen that although residents would check the nutrition label, it does not necessarily affect their shopping behavior, as shown in Table 4.

**Table 4:** Description of variables and summary statistics.

**Variables**	**Items**	**Label**	**Strongly disagree**	**Disagree**	**Modest**	**Agree**	**Strongly agree**
			***N*** **(%)**	***N*** **(%)**	***N*** **(%)**	***N*** **(%)**	***N*** **(%)**
Knowledge	score	Nutrition knowledge score	54 (8.5)	230 (36.2)	229 (36.0)	97 (15.3)	26 (4.1)
	K_K	Understand the information on nutrition labeling	64 (10.1)	130 (20.4)	280 (44.0)	132 (20.8)	30 (4.7)
	k_1	Understand technical term description	203 (31.9)	178 (28.0)	163 (25.6)	64 (10.1)	28 (4.4)
	k_2	Understand the nutrients.	73 (11.5)	103 (16.2)	211 (33.2)	172 (27.0)	77 (12.1)
	k_3	Understand the numerical information and units	134 (21.1)	128 (20.1)	182 (28.6)	119 (18.7)	73 (11.5)
	k_4	Understand the function of nutrients	74 (11.6)	95 (14.9)	192 (30.2)	166 (26.1)	109 (17.1)
Attitude-trust	A_A	Nutrition labeling is credible.	19 (3.0)	111 (17.5)	292 (45.9)	184 (28.9)	30 (4.7)
	a_1	Nutrition Facts Table is credible	33 (5.2)	117 (18.4)	289 (45.4)	173 (27.2)	24 (3.8)
	a_2	Nutrition claims are credible.	32 (5.0)	114 (17.9)	304 (47.8)	159 (25.0)	27 (4.2)
	a_3	Nutrition function claims are credible	32 (5.0)	100 (15.7)	281 (44.2)	186 (29.2)	37 (5.8)
Attitude	a_4	Nutrition labeling can help select healthy foods.	26 (4.1)	82 (12.9)	194 (30.5)	241 (37.9)	93 (14.6)
	a_5	Nutrition labeling is necessary	11 (1.7)	28 (4.4)	113 (17.8)	241 (37.9)	243 (38.2)
Practice	P2	Nutrition labels can affect your shopping behavior	50 (7.9)	119 (18.7)	222 (34.9)	175 (27.5)	70 (11.0)
	p_1	Read nutrient composition and content	65 (10.2)	107 (16.8)	193 (30.3)	189 (29.7)	82 (12.9)
	p_2	Read energy	82 (12.9)	123 (19.3)	213 (33.5)	120 (18.9)	98 (15.4)
	p_3	Check NRV%	112 (17.6)	114 (17.9)	235 (36.9)	117 (18.4)	58 (9.1)
	p_4	Observation nutrition claims	60 (9.4)	102 (16.0)	205 (32.2)	180 (28.3)	89 (14.0)
	p_5	Observe nutrition function claims	62 (9.7)	99 (15.6)	224 (35.2)	152 (23.9)	99 (15.6)

### 5.4. Discriminant validity analysis and testing the fit of the model

Exploratory factor analysis (EFA) examined the factor structure and adjusted the number of items. Pearson's correlation test was used to analyze the relationships among knowledge, attitude, and behavior. The discriminant validity issue was examined by the square root of the average variance extracted (AVE). There was no identification validity problem in this data, as the value of the square root of the AVE was higher than its correlation with other constructs (44), as shown in Table 5.

**Table 5:** Factor correlations and discriminant validity.

**Factors**	**Nutrition knowledge**	**Attitude-trust to nutrition labeling**	**Attitude to nutrition labeling**	**Practice of the nutrition labeling**
Nutrition knowledge	(0.751)			
Attitude-trust to nutrition labeling	0.561^**^	(0.844)		
Attitude to nutrition labeling	0.764^**^	0.429	(0.684)	
Use of the nutrition labeling	0.657^**^	0.329	0.717^**^	(0.790)

Values in brackets () indicate the square root of AVEs. A significance level (^**^p < 0.01).

Kaiser–Meyer–Olkin (KMO) test and Bartlett's test of sphericity (BTS) revealed that KMO=0.914, BTS was significant (χ^2^ = 9,834.497, *p* < 0.001), and the condition of EFA was met, which suggests that these items are suitable for factor analysis (45). The consistency of all scales was tested by composite reliability (CR), and the findings that the average variance extracted (AVE) values exceeded 0.50 for all constructs suggested that the latent constructs retained a minimum of 50% variance. The reliability of the samples was tested by Cronbach's α coefficient, which showed that Cronbach's α = 0.897 for the total scale, and each scale coefficient was >0.83, suggesting good reliability of the questionnaire, as shown in Table 6.

**Table 6:** Factor loadings and convergent validity results.

**Variables**	**Items**	**Standard loadings**	**AVE**	**CR**	**Cronbach's alpha**
Knowledge	Nutrition knowledge score	0.577	0.564	0.883	0.880
	Understand the information on nutrition labeling	0.704			
	Understand technical term description	0.628			
	Understand the nutrients.	0.889			
	Understand the numerical information and units	0.809			
	Understand the function of nutrients	0.845			
Attitude-trust	Nutrition labels are credible.	0.838	0.714	0.909	0.908
	Nutrition Facts Table are credible	0.853			
	Nutrition claims are credible.	0.871			
	Nutrient function claim is credible	0.817			
Attitude2	Nutrition labels can help to choose healthy foods.	0.791	0.468	0.630	0.610
	Nutrition labels are necessary	0.556			
Practice	Reading nutrition labeling when shopping.	0.731	0.625	0.909	0.915
	Reading nutrient composition and content	0.845			
	Reading energy	0.848			
	Check NRV%	0.841			
	Observation nutrition claim	0.761			
	Observe the functional claim of nutrients	0.704			

Rotation technique: Promax; extraction technique: maximum likelihood; Cronbach's alpha=0.922, Kaiser-Meyer-Olkin measure of sampling adequacy: 0.914, p < 0.001, AVE: average variance extracted, CR: composite reliability.

### 5.5. Structural equation modeling fitting for nutrition labels

**Figure 2:**
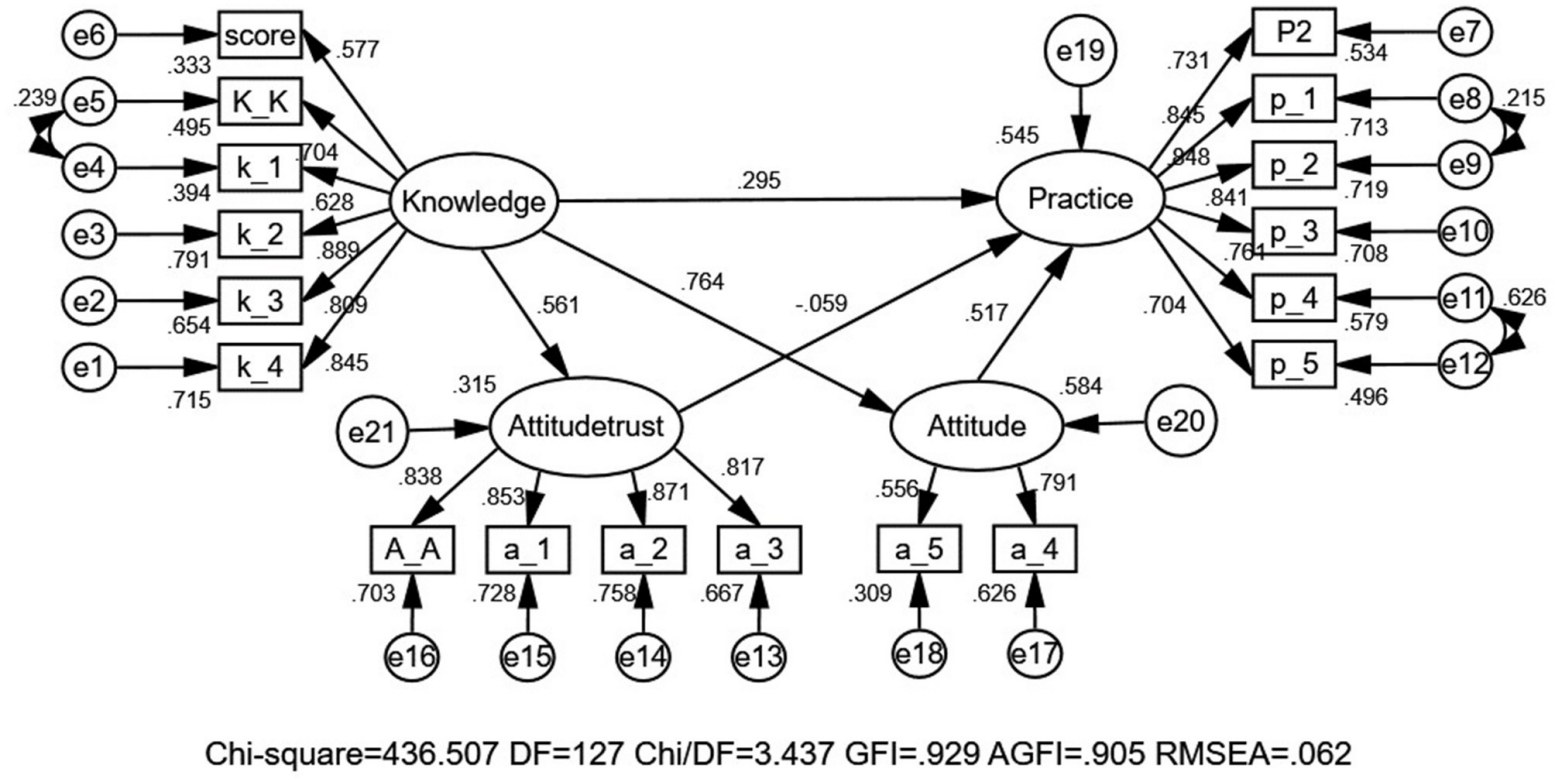
The KAP structural equation model of the nutrition label.

This study investigates whether nutrition knowledge and attitude affect residents' nutrition label use behavior, whether attitude plays an intermediary role between knowledge and use behavior (46), and whether knowledge can directly affect use behavior. We reviewed the relevant references and subdivided the problem of attitude dimension because we find that when all six attitude problems are included in the model, the final model showed unsatisfactory fitness to the data. To solve the problem of unsatisfactory data fitting, we subdivide attitude factors into two potential variables, attitude–trust and attitude, and establish KAP structural equations. First, we established the K-A(trust)-P structural equation. We found that there was only a slight correlation between attitude (trust) and practice, with a path coefficient of 0.003, and the correlation between them was not significant (*p* = 0.941). Then, we established the KAP structural equation model and found that there was a significant positive correlation among knowledge, attitude, and practice, and the path coefficient was > 0. In order to observe the correlation between two attitudes potential variables and practice at the same time. Our research group finally combined the two models together to form a new model (Figure 2). The results showed that the path coefficient from attitude (trust) to practice was −0.059, showing a negative correlation. The path coefficient from attitude to practice was 0.517, and there was a positive correlation.

The model fitted the total samples and explored the relationships among knowledge, attitude, and behavior as latent variables. The fitting index of the structural model (CMIN = 436.507, DF = 127, and CMIN/DF = 3.437 (*p* < 0.05); GFI = 0.929 and AGFI = 0.905; TLI = 0.951, CFI = 0.959, NFI = 0.944, IFI = 0.959, and RMSEA = 0.062) outperformed the respective threshold value, signifying that the data fit the structural model satisfactorily (Table 7).

**Table 7:** Model fitness indices for the modified model.

**Goodness-of-Fit Indices**	**CMIN**	**DF**	**CMIN/DF**	**CFI**	**IFI**	**GFI**	**AGFI**	**NFI**	**TLI**	**RMSEA**
Ideal standards			< 5.0	>0.90	>0.90	>0.90	>0.90	>0.90	>0.90	< 0.08
Measurement value	436.507	127	3.437	0.959	0.959	0.929	0.905	0.944	0.951	0.062

CMIN/DF, Chi-square fit statistics/degree of freedom; CFI, comparative fit index. IFI, incremental fit index; GFI, goodness-of-fit index; AGFI, adjusted goodness-of-fit index; NFI, Normed Fit Index; TLI, Tucker-Lewis index; RMSEA, root mean square error of approximation.

As shown in Figure 2, Table 8. Hypothesis 1: The path coefficient from knowledge to attitude–trust is 0.561 (*p* < 0.001), which indicates that residents' nutritional knowledge level is positively and significantly associated with their trust. Hypothesis 2: The path coefficient from knowledge to attitude is 0.764 (*p* < 0.001), which indicates that residents' nutritional knowledge level is positively and significantly associated with their attitudes. Hypothesis 3: The path coefficient from knowledge to practice is 0.295 (*p* = 0.001), which indicates that residents' nutrition knowledge will directly impact their use of nutrition labeling, with a positive significant correlation. Hypothesis 4: The path coefficient from attitude–trust to practice is −0.059(*p* = 0.171), indicating that residents' trust in nutrition labeling was inversely related to practice, and the path coefficient was not insignificant. Hypothesis 5: The path coefficient from attitude to practice is 0.517 (*p* < 0.001), which indicates that residents' nutrition attitude will impact their use of nutrition labeling, with a positive significant correlation. Thus, hypothesis 5 indicated that attitude played a greater role as an indirect effect between knowledge and behavior, while hypothesis 4 indicates that trust limits residents' practice of nutrition labeling. It could be explained that nutrition knowledge was the prerequisite for label reading behavior, and attitude was the intermediary effect.

**Table 8:** Test results of the hypothesis.

**Hypothesized paths**	**Normalized path coefficient**	***T* value**	**Accepted**
H1:Nutrition knowledge → Attitude-trust to the nutrition labeling	0.561^***^	13.101	YES
H2:Nutrition knowledge → Attitude to the nutrition labeling	0.764^***^	16.503	YES
H3: Nutrition knowledge → practice of nutrition label.	0.295^*^	3.291	YES
H4: Attitude-trust to the nutrition labeling → practice of nutrition label.	−0.059	−1.368	NO
H5: Attitude to the nutrition labeling → practice of nutrition label.	0.571^***^	4.914	YES

Levels of statistical significance (^***^p < 0.001, ^*^p < 0.05).

## 6. Discussion

The results of this research indicated that the overall cognition level of community residents on nutrition knowledge was low, with an awareness rate of 32.7%, which was unsatisfactory and lower than the national average level (47). Residents have a positive attitude toward nutrition labeling. Approximately 76.1% of the residents indicate that it was necessary to mark the nutrition label on the food packaging; 52.5% of the residents believed that nutrition labeling could help healthy eating or shopping choices in the future, and 33.6% of the residents considered that the nutrition labeling was credible. In total, 38.5% of participants indicated that nutrition labeling would affect their shopping behavior. However, only 25.3% of the residents could understand nutrition labeling, indicating that most of the residents had a positive attitude toward nutrition labeling, but they lack a correct understanding of nutrition labeling and doubt their authenticity. The main reason may be that the promotion of nutrition labeling in China is done mainly to increase the reliability and marking rate of labels, rather than educating residents on nutrition knowledge popularization, label content interpretation, and use training.

Previous studies have shown that there are still existing obvious gaps between the identification of nutrition labeling and use behavior in real life. Especially, young people who are active consumers of prepackaged foods, have plenty of chances to contact with nutrition labeling but rarely use them effectively in fact. The practice of nutrition labeling not only depends on whether to establish health belief and implement restaurant menu labeling (48, 49) but also depends on demographic, social, and psychological factors of the population. (50). In this study, we found that residents who were young, female, having high education level, and having high socioeconomic status had higher awareness of nutrition labeling and more positive attitudes, and the frequency of checking nutrition labeling is also higher, which was consistent with previous studies (51, 52). With increasing attention to weight loss, calorie intake restriction, and own health in recent years, nutrition labeling can be an effective tool to directly obtain the nutrition information of packaged food for consumers, which can also help consumers to make a healthy choice. Therefore, nutrition labeling plays an indispensable role in helping residents maintain healthy eating habits (53).

The advantage of this study is its adoption of the mature KAP model to analyze Chinese community residents' cognitive status of nutrition labeling, which was divided into knowledge, attitude, and practice, and then establishing the structural equation. Regarding statistical methodological strength, structural equation modeling is superior to multiple linear regression modeling. The structural equation model can analyze multiple dependent variables at the same time, and its application is helpful to scientifically analyze the relationship between indicators. In this study, SEM is helpful to analyze the direct effects of the nutrition label KAP and to reveal these relationships. However, there are still a few limitations in the present study. First of all, more rigorous survey questions need to be designed. For example, participants were likely to make inaccurate responses, since the nutrition labeling contains a lot of information and residents are likely to confuse the list of ingredients with the nutrition fact table. Furthermore, we only used one topic to assess the residents' previous nutrition education experience, and we also tested the residents' subjective knowledge. In future research, we need more objective scales to measure residents' subjective knowledge of nutrition labeling, rather than using simple self-reporting questions, rather than through the use of simple self-reported questions. Finally, our sample size was small, drawn by the convenience sampling method, and hardly ensured that the findings above could be replicated within behavioral studies. Other mediating factors (e.g., peer or parental impact on their use of nutrition labeling, understanding of diet-related disease information, taste or sensory attributes of the product) might more effectively explain that residents' use of nutrition labeling was not included in the study and need to be explored in future studies.

Behavior changes of community residents were divided into three continuous processes: knowledge acquisition, belief generation, and behavior formation, which are positive relations (path coefficients > 0). In this study, the path analysis demonstrated that the path coefficient between nutrition labeling knowledge and trust was 0.561 (*p* < 0.05), and the path coefficient between nutrition labeling knowledge and attitude was 0.764 (*p* < 0.05), with a significant positive correlation between them, indicating that residents could form a more positive attitude toward the nutrition labeling if they were knowledgeable about the nutrition labeling. Evelyn et al. (54), Rimpeekool et al. (55), Jackey et al. (56), and Cannoosamy et al. (57) also reported similar results in their respective investigations.

Previous studies have suggested more nutrition knowledge, and health-motivated residents might be more skeptical about nutrition claims and nutrition function claims, thus limiting residents' practice of nutrition labeling. We also tested this relationship, and the correlation analysis found that there was a significant positive correlation between nutrition knowledge and trust (path coefficient = 0.561, *p* < 0.05), the trust was negatively correlated with nutrition practice, but it was not significant (path coefficient = −0.059, *p* > 0.05), which may be the most residents are skeptical about the authenticity of nutrition labeling in this study. Residents' trust score is low, which leads to a negative correlation between trust and the practice of nutrition labeling, which was the same as the previous study.

We found that more nutrition knowledge and positive attitudes could increase the practice of nutrition labeling among residents in this research, which was consistent with the results of previous studies (58–61). It means that based on the model, consumers are likely to establish positive beliefs, and finally change use behaviors, once they receive nutrition education (55). However, it should be noted that the residents' trust in nutrition labeling was not significant with their frequency of using nutrition labeling. Therefore, in this study, attitude is a psychological reaction (including helpfulness and necessity) to convince ourselves that nutrition labeling is helpful and useful to select healthy foods, which will further change our practice of nutrition labeling (62, 63).

In summary, we confirmed that residents' nutrition knowledge could be directly converted into nutrition labeling reading behavior or indirectly through changing their attitudes. Residents with higher nutrition labeling knowledge scores and more positive attitude towards nutrition labeling seem to be more likely to obtain the information provided on nutrition labeling. It reflected that knowledge of nutrition was the basis for changing the practice of nutrition labeling. Rich nutrition knowledge can promote the use of nutrition labeling, while poor nutrition knowledge will limit their practice. Therefore, we must pay attention to the synchronous development of nutrition labeling KAP.

## 7. Conclusion and recommendations

The KAP model is suitable for analyzing the use behaviors of nutrition labeling by local residents. There was a direct and indirect correlation between nutrition knowledge and the practice of nutrition labeling. The attitude of nutrition labeling was positively affected by knowledge, while the use behavior of nutrition labeling was positively affected by knowledge and attitude.

To improve the lifestyle of residents and correctly use nutrition labeling, the following policy recommendations are offered. First, more public education programs (e.g., printing graphic brochures or posters, learning websites, and special lectures) should be implemented in schools and communities. The purpose of public education programs is to make the public aware of “the availability of nutrition information in nutrition labeling and the importance of that information in maintaining healthy dietary practices” to improve their nutrition literacy. Specifically, the interpretation of nutrition labeling needs to be included in the *Dietary Guidelines for Chinese Residents* and should be disseminated in the annual National Nutrition Week activity. The theme of National Nutrition Week 2022 is Learn How to Cook, How to Select Ingredients Reasonably, and Check Nutrition Labeling. The guideline, “Learn to read food labeling and choose prepackaged foods reasonably”, highlights the core value of “Check Nutrition Labeling”. Second, the concept and function of NRV% and core nutrients on the nutrition facts table, especially sodium and fat, should be conveyed transparent. It is suggested to mark NRV% explanation on food packaging to ensure consumers understanding. Then, it is necessary to strengthen the nutrition education of residents so that they fully understand the meaning of nutrition claims and nutrient function claims and avoid confusing nutrition function claims and health food function claims.

Finally, it will appeal to the relevant departments to implement effective supervision and inspection to ensure the accuracy of nutrition labeling information, which can in turn enhance consumers' confidence in the nutrition labeling. With the government as the leading role and the participation of the whole society, we should strengthen the publicity and education of labeling knowledge and improve residents' nutritional literacy and cognitive attitude toward labeling knowledge to change their behaviors.

## Data availability statement

The raw data supporting the conclusions of this article will be made available by the authors, without undue reservation.

## Ethics statement

The studies involving human participants were reviewed and approved by Ningxia Medical University Ethics Committee. The patients/participants provided their written informed consent to participate in this study.

## Author contributions

JY conceived and designed this research and revised the manuscript. YL was responsible for data analyses and prepared the manuscript. Both authors have read and approved the final version.
