# Supplementary material for: Status of nutrition labeling knowledge, attitude, and practice (KAP) of residents in the community and structural equation modeling analysis
Source: Front Nutr. 2023 Apr 17;10:1097562. doi: 10.3389/fnut.2023.1097562 (PMC10149812; doi:10.3389/fnut.2023.1097562)
Supplement: Supplementary file 1 [file Data_Sheet_1.PDF]

(Appendix)

Investigation and Research on Community Residents' Cognition of Nutrition Labeling in prepackaged foods

Number: _____

Dear Citizens：

We are investigators from School of Public Health and Management, Ningxia Medical University. We are conducting a survey on residents' knowledge, attitude and behavior towards nutrition labeling. The research results will help to promote the development of food nutrition labeling in China. This study uses the self-filled questionnaire, which needs to take you some time. Your answer is not right or wrong，please fill in the answer objectively and truthfully. Each choice you make will provide us with valuable research content!

The following principles will be followed during the study:

1.Voluntary participation in the study

2.Respect your right to privacy, adopt anonymous investigation and strictly keep all relevant information received confidential.

3.You have the right to know the results of this study.

4.You have the right to withdraw from the study at any time.

Hope to get your cooperation! Thank you for your support first!

School of Public Health and Management

Ningxia Medical University

Investigator: Investigation date:

Checker: Verification date:

Basic information（A）

Description of frequency level：

Always (8 times or more out of 10) **often (6-7 times out of 10)** **occasionally (3-5 times out of 10)**

rarely (1-2 times out of 10) **never (none at all)**

1.Gender：①Male　　②Female

2.Your age：_______years old

3.Your highest education (including currently studying) is：

①Primary school and below　　②Junior Secondary

③Senior /Technical Secondary or vocational high

④Undergraduate or tertiary　　⑤Postgraduate or above

4.Your current occupation：

①Government and institution staff　　②Staff of state-owned enterprises

③Staff of private enterprises　　④Farmers or workers

⑤ Self-employed　　⑥ Students　　⑦ Temporarily unemployed

⑧ Retired　　⑩Other occupations _____________

5.Your current marital status：

①Unmarried　②Married　③Divorced　④Widowed

6.Your total monthly income：

①≤1,500 yuan　　②1,501 - 3,000 yuan　　③3,001-5,000yuan

④5,001-10,000yuan　　⑤10,001 -20,000yuan　　⑥≥20,000yuan

7.Your current financial pressure:

①Very small　　②Relatively small　　③Neither big nor small

④Relatively large　　⑤Great

8.Do you or any member of your family suffer from any of the following illnesses? (Multiple choices are allowed)

①Hypertension　　②Cardiovascular disease　　③Diabetes

④Overweight or Obesity　⑤Osteoporosis　　⑥Hyperlipoidemia

⑦Hypercholesterolemia　⑧Fatty Liver　　⑨Chronic Nephritis

⑩Food Allergy　　⑪No diseases mentioned above.　⑫Others________

9.In the past year, how do you feel your health：

①Very bad　　②Relatively bad　　③Neither bad nor good

④relatively good　　⑤very good

10.Have you received medical or nutrition-related professional knowledge education

or training？

①Yes ②No

11.Do you undertake purchasing/cooking tasks in your home?

① Never ②Rarely ③Occasionally ④Often ⑤Aways

Nutrition Knowledge（B）

1.Do you know the“**Dietary Guidelines for Chinese Residents**”:

①Yes ②No

2.How do you get your **Nutrition Knowledge**？（Multiple choice are allowed）

①Classmates, colleagues, friends ②Family or relatives

③Training/classes/lectures ④Television broadcast/internet

⑤Books/newspapers/magazines

3.Do you know what the main function of **carbohydrates** (rice, noodles, bread) is？

①The main source of energy ②Antioxidation

③Promote that growth of organism tissue ④Enhance immunity.

4.Do you know what the main function of **protein** (egg, soybean, milk) is？

①Replenish the brain energy, anti-fatigue ②Enhance immunity

③Promote growth and metabolism ④Prevent chronic diseases

5.Do you know what the main function of **fat** (nuts, fats) is？（Multiple choice）

①Provide energy and maintain body temperature

②Increase Satiety

③Provide human Essential fatty acid

④Auxiliary fat-soluble vitamin absorption

6.Do you know what the following consequences will be caused by the intake of high energy and high fat (fried chicken, hamburgers, cakes)? (Multiple choice）

①Overweight/obesity

②It is easy to cause cardiovascular disease

③It is easy to cause cancer

④ It is easy to cause diabetes

7.Do you know the main function of **dietary fiber** (whole grains, vegetables, fruits) ?

（Multiple choice）

①Increase intestinal peristalsis

②Reduce blood glucose and cholesterol

③Increase intestinal beneficial bacteria

④Maintain normal physiological function of intestine

8.Do you know what **cholesterol** is?

①It is harmful to human body, is the "accomplice" of cardiovascular and cerebrovascular diseases

②It is an important component of the cell membrane

③As long as you do not take in cholesterol, the human body will not produce

④Children and teenagers should not eat foods containing cholesterol.

9.Which of the following dietary patterns can reduce the incidence of cardiovascular disease in residents：

①Oriental dietary pattern

②Dietary patterns in economically developed countries

③Japanese dietary patterns

④Mediterranean dietary patterns

⑤The dietary pattern of our country

10.High blood pressure is associated with excessive intake of any of the following nutrients？

①Protein ②Vitamin ③Potassium ④Sodium ⑤I don’t know

11.How many grams of salt does "*Dietary Guidelines for China Residents 2022*" recommend for adults to eat daily?

①3 g ②5 g ③6 g ④8 g ⑤I don’t know

12Low sodium refers to how many milligrams per 100g of solids /100ml of liquid food does not contain more than sodium?

① 80mg ②100mg ③120mg ④150mg ⑤I don’t know

Nutrition labeling（C）

1. When you buy food, what do you usually see on the packaging?

①List of food ingredients

②Table of nutrient composition

③Production date and shelf life

④Manufacturers and brands

2.Do you know/understand the nutrition labeling?（**Usually appears on the back of food packaging**）

①I have no idea about it ②I don’t know much about it ③It's hard to explain

④I know it ⑤I know it quite well

3.Do you think it is necessary to put nutrition labeling on food package?

①Not at all ②Not really ③It's hard to explain

④ Necessary ⑤Very Necessary

4. What parts do you think the food nutrition labeling contains?

①Food name, food ingredient list, method of eating

②Food production date, shelf life, storage conditions

③Food Nutrition Facts Table, nutrition claims and nutrition function claims

④Batch number of food production, name and address of production enterprise

5.What are the following core nutrients that are mandatory on the nutrition labeling in prepackaged foods?

①Energy, carbohydrates, vitamins, minerals, sodium

②Energy, fat, protein, carbohydrates, sodium

③Fat, energy, protein, vitamins, calcium

④Fats, minerals, proteins, vitamins, sugars

6.What is the meaning of nutrient reference values (NRV) in the food nutrition labeling?

①It refers to the reference intake standard of dietary nutrients for residents in China

②It refers to the reference standard specially used for comparing the content of food

nutrients on the food nutrition label

③It refers to the level that the daily intake of nutrients for residents in China must reach.

④Nutrients reference standards applicable to all people.

7.7. Do you think the information on food nutrition labeling is easy to understand?

①not at all ②Not So Easy ③Uncertain ④easy to understand ⑤Very easy

8.How well do you understand the content of nutrition labels? （Please tick '√'in the box.）

Content	Not at all	Poor understand	Uncertain	Mostly understand	completely understand
Technical term description (e. g. NRV%)					
Types of nutrients (e. g. protein, fat, etc.)					
numerical information and units (e. g.g/mg/kJ)					
The function of nutrient components (e. g. Calcium contributes to bone health)					

9. Do you think the nutrition label is credible?

①Completely unreliable ②Not very credible

③Uncertain ④Credible ⑤Completely credible

10.How credible do you think the content of the nutrition labeling is?（Please tick '√'in the box.）

Content	Completely unreliable	Not very credible	Uncertain	Credible	Completely credible
Nutrient facts table (nutrient composition/content/NRV%)					
Nutrition claims (e.g. , low sugar, high protein, etc.)					
nutrition function claims (e.g. calcium helps increase bone density)					

11.Do you think the nutrition labeling will help you with your healthy eating habits

and shopping choices in the future?

①Completely unhelpful. ②Not much help. ③Uncertain ④Helpful

⑤Completely helpful

12. When you buy food, do you check the information on the nutrition labeling **(ingredient list, nutrition fact table, etc.)**?

①Never ② Rarely ③Occasionally ④Often ⑤ Always

13. What are the reasons that affect you to check the nutrition label to choose food?（Please tick '√'in the box.）

Cause	Completely no influence	Not much influence	Uncertain	Influence	Completely influence
The nutrition labeling has many contents and wastes time.					
The nutrition labeling is too complicated to read					
Nutrition labeling content false, not worth reading					
Don't care about nutrition, as long as it tastes good					
Interested in nutrition labels					

14. Does the nutrition label affect your shopping behavior?

①Never ②Rarely ③Occasionally ④Often ⑤Always

15. What information in the Nutrition label will affect your shopping choices?（lease type in the box to the degree"√"）

Cause of effect	Completely no influence	Not much influence	Uncertain	Influence	Completely influence
The type and content of a nutrient (fat, protein, etc.)					
Energy/calories					
NRV%					
Nutrition claims (e.g., low sugar, high protein, added vitamin C)					
Nutrition function claims (e.g.Calcium helps improve bone density, dietary fiber benefits digestive system health)					

------Thank you for your cooperation！------
